# Commentary: Playing the computer game tetris prior to viewing traumatic film material and subsequent intrusive memories: examining proactive interference

**DOI:** 10.3389/fpsyg.2016.00260

**Published:** 2016-02-23

**Authors:** Angelica B. Ortiz de Gortari, Mark D. Griffiths

**Affiliations:** International Gaming Research Unit, Psychology Division, Nottingham Trent UniversityNottingham, UK

**Keywords:** post-traumatic stress disorder, visual intrusions, video Games, game transfer phenomena, trauma prevention, intrusive memories

Unwanted visual intrusions are characteristic of Post-Traumatic Stress Disorder (American Psychiatric Association, 2013). One innovative intervention for inhibiting unwanted intrusions is playing the *Tetris* videogame, described as a “cognitive vaccine” in preventing intrusions after traumatic events (Holmes et al., 2009). Playing *Tetris* consumes heavy visuospatial working memory resources that potentially compete with cognitive resources required for elaboration of visual imagery. Since Holmes et al.'s (2009) study, other studies have used *Tetris* to inhibit intrusive imagery (Holmes et al., 2010; Skorka-Brown et al., 2014, 2015; James et al., 2015a,b). However, these have not assessed the role of videogame content after playing in relation to Game Transfer Phenomena (GTP) (Ortiz de Gortari and Griffiths, 2014a,b,c, 2015a,b).

GTP research has investigated non-volitional experiences (e.g., altered sensorial perceptions and automatic mental processes/behaviors) mostly experienced after gaming. Gamers often report sensorial (visual/auditory) intrusions after playing (e.g., visual and auditory imagery, hallucinations) (Ortiz de Gortari et al., 2011; Ortiz de Gortari and Griffiths, 2014a,b). In a survey of 2362 gamers, most (77%) had visualized images from a variety of videogames (including tile-puzzle games) with closed-eyes, and one-third (31%) had visualized images with open-eyes (Ortiz de Gortari and Griffiths, 2015b). Other studies have experimentally induced videogame-related visualizations at sleep onset (Stickgold et al., 2000; Wamsley et al., 2010; Kusse et al., 2012).

James et al. (2015a,b) study was the first to make explicit reference to GTP (referred to as the “*Tetris* effect” [TE]). Ortiz de Gortari and Griffiths (2012) argued the TE term is misleading as it suggests repetition is the core of transfer effects. However, other factors are involved. Research concerning GTP makes the distinction between sensorial modalities facilitating the assessment of non-volitional phenomena with videogame content that occur along the continuum from mild to severe (Ortiz de Gortari and Griffiths, under review). Moreover, the descriptive constructs of GTP are empirically based on analysis of 3500 + gamers and have been examined via confirmatory factor analysis demonstrating good reliability and validity (Ortiz de Gortari et al., 2015).

James et al. (2015a,b) tested if playing *Tetris* offered a protective mechanism against re-experiencing traumatic events. Healthy participants (*n* = 56) were randomly assigned to either playing *Tetris* for 11 min, or doing nothing before exposure to a 12-min traumatic film. Image-base memories about the film were then registered in a 1-week dairy. However, playing *Tetris* as a proactive interference task before watching the film did not show significant results. James and colleagues offered different explanations including: (i) duration of the task in relation to film length, (ii) temporal contingencies between the tasks, (iii) differences between the task types, (iv) videogame types used, and (v) reactivation of gameplay during the film for aided interference. These explanations are discussed in relation to GTP literature.

## Duration of task in relation to film length

Playing *Tetris* for 11 min may not have been long enough to compete with the consolidation of memory of the 12-min film. GTP are significantly more likely to occur when playing 3–6 h. Only 4% of gamers reported GTP when playing sessions shorter than 1-h (Ortiz de Gortari and Griffiths, 2015a). Laboratory experiments have taken days of playing to induce game-related visualizations at sleep onset (Stickgold et al., 2000; Wamsley et al., 2010; Kusse et al., 2012).

## Temporal contingencies between gaming and film watching

The tasks were performed minutes apart from each other. GTP mostly occurs soon after stopping playing but gamers have also reported GTP days after playing (Ortiz de Gortari and Griffiths, 2014c). In most cases, duration of experience is very short (seconds/minutes) but in some cases hours or longer.

## Differences between the tasks

Previous studies have suggested that similar tasks aid interference (Bunting, 2006). Realistic videogames may reduce the differences between the tasks. Since playing videogames requires additional perceptual/motor skills than watching a passive film, it may be that gaming is more potent as an interference task, particularly if the subjective sense of presence in the virtual world is induced.

## Type of videogame used as interference task and emotional content of film

The unrealistic (geometric) *Tetris* content may have been overwritten by the film's traumatic images. Visualization of stereotypical games induced at sleep onset are characterized by lack of emotion, assuming that the amygdala and the reward system are not involved (Kusse et al., 2012). In GTP research, emotions in tile-matching puzzle-games are incomparable to emotions in realistic videogames (Ortiz de Gortari and Griffiths, 2014a).

## Reactivation of gameplay during the film for aided interference

The use of cue reminders may have potential in reviving videogame content. In many cases, thoughts and altered perceptions are triggered by game-related cues (Ortiz de Gortari and Griffiths, 2014a). Attentional bias toward game-related cues has been demonstrated in experiments (Metcalf and Pammer, 2011). GTP have been reported in variety of videogame genres particularly those that have very realistic graphics and settings. Therefore, more realistic games may aid associations between real life stimuli and videogame content, and may be more effective in competing with memories of traumatic events.

## Conclusion

In summary, playing *Tetris* is not only an effective visuospatial task (overloading working memory resources needed for imagery-formation while playing), but as demonstrated in GTP studies, videogame content stays active after playing (e.g., mental imagery, sensory perceptions), and may offer additional benefits for managing unwanted intrusions. GTP may potentially strengthen effects of interference tasks but should be used cautiously, because videogame content not only targets unwanted intrusions, but also influences individual cognitions, perceptions, and behaviors in day-to-day contexts (e.g., attention bias, lack of task awareness, control inhibition failures) (Ortiz de Gortari and Griffiths, 2015a). Moreover, distress and dysfunction have been reported with GTP (Ortiz de Gortari and Griffiths, 2015b, under review).

Further, research needs conducting to identify: (i) videogames that are most effective, (ii) playing duration, (iii) factors that reduce intervention efficacy and strategies to control them, and (iv) individuals that may benefit the most from such intervention. While using videogames as intervention tools for preventing unwanted imagery from traumatic experiences has potential, therapeutically it is still at an early stage.

## Author contributions

AO had the initial idea and wrote the first draft of the paper. Both authors revised the paper critically, edited, and agree on the final version. MG edited down the final draft version.

### Conflict of interest statement

The authors declare that the research was conducted in the absence of any commercial or financial relationships that could be construed as a potential conflict of interest. The reviewer, KP-T, and handling Editor declared their shared affiliation, and the handling Editor states that the process nevertheless met the standards of a fair and objective review.
